# Knowledge, attitude and techniques of breastfeeding among Nigerian mothers from a semi-urban community

**DOI:** 10.1186/1756-0500-6-552

**Published:** 2013-12-21

**Authors:** Chidozie E Mbada, Adekemi E Olowookere, Joel O Faronbi, Folasade C Oyinlola-Aromolaran, Funmilola A Faremi, Abiola O Ogundele, Taofeek O Awotidebe, Adepeju A Ojo, Oluwakemi A Augustine

**Affiliations:** 1 Department of Medical Rehabilitation, College of Health Sciences, Obafemi Awolowo University, Ile – Ife, Nigeria; 2 Department of Nursing Sciences, Obafemi Awolowo University, Ile – Ife, Nigeria

## Abstract

**Background:**

Mothers’ poor knowledge and negative attitude towards breastfeeding may influence practices and constitute barriers to optimizing the benefits of the baby-friendly initiative. This study assessed breastfeeding knowledge, attitude and techniques of postures, positioning, hold practice and latch-on among Nigerian mothers from a Semi-Urban community.

**Methods:**

Three hundred and eighty three consenting lactating mothers who have breastfed for 6 months and up to two years volunteered for this cross-sectional survey, yielding a response rate of 95.7%. A self-administered questionnaire that sought information on maternal socio-demographic variables, knowledge, attitudes and breastfeeding techniques of mothers was employed.

**Results:**

Based on cumulative breastfeeding knowledge and attitude scores, 71.3% of the respondents had good knowledge while 54.0% had positive attitude. Seventy one point three percent practiced advisable breastfeeding posture. Sitting on a chair to breastfeed was common (62.4%); and comfort of mother/baby (60.8%) and convenience (29.5%) were the main reasons for adopting breastfeeding positions. Cross-cradle hold (80.4%), football hold technique (13.3%), breast-to-baby (18.0%) and baby-to-breast latch-on (41.3%) were the common breastfeeding techniques. A majority of the respondents (75.7%) agreed that neck flexion, slight back flexion, arm support with pillow and foot rest was essential during breastfeeding. There was no significant association between breastfeeding posture practice and each of cumulative breastfeeding knowledge score levels (X^2^ = 0.044; p = 0.834) and attitude score levels (X^2^ = 0.700; p = 0.403).

**Conclusion:**

Nigerian mothers demonstrated good knowledge and positive attitude towards breastfeeding. Most of the mothers practiced advisable breastfeeding postures, preferred sitting on a chair to breastfeed and utilized cross-cradle hold and baby-to-breast latch-on.

## Background

Breastfeeding is the act of milk transference from mother to baby [1] that is needed for the survival and healthy growth of the baby into an adult [2,3]. Breastfeeding creates an inimitable psychosocial bond between the mother and baby [4,5], enhances modest cognitive deve lopment [6] and it is the underpinning of the infant’s wellbeing in the first year of life [5,7] even into the second year of life with appropriate complementary foods from 6 months [8]. Furthermore, breastfeeding reduces the risk of neonatal complications [9], respiratory and other varieties of illnesses [10-13].

Based on anecdotal and empirical evidence on the benefits of breastfeeding to the mother and baby, the World Health Organization (WHO) [8] has recommended 2 year breastfeeding; first 6 months exclusive breastfeeding; more than 8 times breastfeeding of the baby per day in the first 3 months of an infant’s life. The WHO and the United Nations Children’s Fund (UNICEF) global effort to implement practices that protect, promote and support breastfeeding through the Baby-Friendly Hospital Initiative has recorded attendant successes [14]. However, a gamut of factors not limited to race and cultural beliefs [15,16], maternal characteristics [17,18], infant health problems [17], socio-economic status [19,20] and some psychosocial factors [21,22] may hamper the full realization of the baby-friendly initiative. Information about the beliefs and knowledge that may constitute barriers and in turn influence practices are needed in order to optimally utilize the benefits of the baby-friendly initiative. Consequently, a number of studies have assessed knowledge, attitude and practice of breastfeeding in different parts of the world; however, such studies are limited among Nigerian mothers [23-26]. Furthermore, there is an apparent dearth of empirical data on breastfeeding techniques among nursing mothers in sub-Sahara Africa (SSA). This study assessed breastfeeding knowledge, attitude and techniques of postures, positioning, hold practice and latch-on among Nigerian mothers.

## Methods

A cross-sectional survey of lactating mothers who had breastfed for not less than six months and up to two years was carried out. These mothers who were attending post-natal clinics for immunization were recruited using consecutive sampling technique. Five government-owned post-natal clinics namely Ife Hospital Unit of the Obafemi Awolowo University Teaching Hospital Complex (OAUTHC), Urban Comprehensive Health Centre (UCHC) of the OAUTHC, Eleyele, Enuwa Primary Health Center, Comprehensive Health Center Aderemi and Okeitubu Comprehensive Health Center Modakeke in Ile-Ife, were purposively selected based on the availability and practice of the baby-friendly programmes in the facilities. Ile – Ife is ancient semi-urban settlement and the traditional home of the Yoruba civilization. Ile-Ife is located in the south-western part of Nigeria bordered by Ibadan (the largest city in SSA) on the West and by Ondo town on the east.

Ethical approval for the study was sought from the Ethics and Research Committee of the OAUTHC. All the respondents gave signed informed consent prior to participation. The questionnaire for the study was adapted from previous studies on knowledge, attitude and practice of breastfeeding [27-29]. The face validity of the questionnaire was determined by expert review in a pilot study. The reliability of the questionnaire was tested by a test-retest method. The copies of the questionnaire were first administered to 20 breastfeeding mothers attending the UCHC and were re-administered 7 days after. The questionnaire was translated into Yoruba language and back-translated into the original language (English). The reliability of the Yoruba version was assessed in a pilot study by a test-retest method among 20 mothers attending the UCHC, observing 7 days between test and re-test. Test-retest reliability of the English version of the questionnaire yielded a correlation coefficient of r = 0.967 at p = 0.001. The items of the Yoruba language (the local language among the respondents) version of the questionnaire yielded agreements percentage that ranged from 94 to 100%, and the corresponding kappas ranged from 0.76 to 1.00.

The self-administered questionnaire sought information on socio-demographic and maternal characteristics, knowledge and attitude towards breastfeeding, and the most usual breastfeeding techniques of postures, positioning, hold practice and latch-on. Knowledge and attitude regarding breastfeeding were rated on a likert scale of 1 to 5 and was transformed to a 100 point scale by multiplying by 20. A pictorial assessment of breastfeeding postures was also included in the questionnaire.

### Data analyses

Data on maternal socio-demographic characteristics, breastfeeding awareness, postures, positioning, hold practice and latching techniques were summarized using descriptive statistics of frequency and percentages. The respondents rating of questionnaire items regarding knowledge and attitude about breastfeeding were summarized using mean and standard deviation. Chi-square test was used to compare each of breastfeeding knowledge, attitude and position practices based on parity and history of previous training on breastfeeding. Also chi-square was used to test association between breastfeeding position practice and each of cumulative breastfeeding knowledge score levels and attitude score levels. The level of significance was set at p < 0.05. Data were analyzed with Statistical Package for Social Sciences (SPSS) software (version 16).

## Results

A total of 400 questionnaires were distributed to breastfeeding women. However, 383 were returned and found valid for analysis yielding a responsive rate of 95.7%. The maternal socio-demographic characteristics are presented in Table 1. The mean age of the respondent was 29.0 ± 4.96 years. Information on breastfeeding awareness among the respondents is presented in Table 2. A majority (88.0%) of the respondents reported to have heard about Exclusive Breastfeeding (EBF) and hospital was the source of information respectively. More than half (69.5%) of the respondents had previous EBF training. Table 3 shows knowledge and attitude of the respondents about breastfeeding. On a scale of 100, knowledge item ‘breastfeeding promote mother-baby bonding’ was scored 76, indicating a high agreement, while item ‘three month of breastfeeding is long enough’ had the lowest score of 36. Regarding attitude to breastfeeding, on a scale of 100, items ‘breastfeeding is easier than feeding infant on formula’ ‘community encourages breastfeeding over feeding infant formula’ and ‘doctors and nurses encourage breastfeeding’ were scored 76 respectively. On the other hand, ‘breastfeeding has effect on care of other family members and on marital relationship’ was the least rated with a score of 42.

**Table 1:** Maternal socio-demographic characteristics (N = 383)

**Variable**	**n**	**%**
Religion		
Christianity	275	71.8
Islam	108	28.2
Education		
Pry	33	8.6
Secondary	157	41.0
Tertiary	185	48.3
MSC	8	2.1
Occupation		
Unemployed	62	16.2
Private unskilled	157	41.0
Private skilled	76	19.8
Public junior	62	16.2
Public senior	26	6.8
Age (in years)		
≤ 30	224	58.5
> 30	159	41.5

**Table 2:** Information on breastfeeding awareness among the respondents

**Variable**	**n**	**%**
Heard about EBF?		
Yes	378	88.0
No	5	1.3
EBF information source		
Hospital	337	88.0
Others	46	12.0
Have you received any training on EBF?		
Yes	266	69.5
No	117	30.5
How many years should a baby breastfeed?		
6 months	3	0.8
Within one year	86	22.5
Within 18 months	168	43.9
Up to 2 years	126	32.9

**Table 3:** Knowledge and attitude about breastfeeding

**Item**	**Average score/(100%)**
KNOWLEDGE	
1. Woman who is fully breastfeeding is less likely to become pregnant 3 months after delivery than a woman who is formula feeding	48
2. Breastfeeding decreases diarrhea	46
3. Breastfeeding is a good contraceptive method	48
4. Breastfeeding promote mother-baby bonding	76
5. Feeding infant formula keeps the body well shaped and prevent over weight	70
6. Frequent breastfeeding in the early period can help reduce jaundice	66
7. Growth pattern of breastfed infants differ from those of formula fed	66
8. If a breastfed infant has not regained his birth weight by two weeks of age the mother should be encouraged to begin supplementing with formula.	62
9. Initial breast production of yellow water (colostrums) is nutritionally useless for the baby and should be discarded	52
10. Supplemental feeding is detrimental to the establishment of a good milk supply	48
11. Three month of breastfeeding is long enough	36
12. Work places provide designated areas for breastfeeding	36
ATTITUDE	
1. Breastfeeding has effect on care of other family members and on marital relationship	42
2. Breastfeeding is a good way to decrease family expenses	72
3. Breastfeeding is easier than feeding infant on formula	76
4. Community encourages breastfeeding over feeding infant formula	76
5. Doctors and nurses encourage breastfeeding	76
6. Feeling shy of breastfeeding in public places	74
7. I’m not comfortable with breastfeeding	54
8. It is not difficult for breastfeeding mother to care for family	62
9. It is usually advisable for babies to receive a formula feed before the first breastfeed	50
10. Maternity leave of 3 months is enough to successful breastfeeding	48
11. Medical practitioners have no role in breastfeeding	48
12. Mother of an infant who feels that she has insufficient milk should top up with, a bottle after each feed.	56

Table 4 show results on the breastfeeding postures, positioning, hold and latching practices. 71.3% of the respondents practiced advisable breastfeeding positioning. More than half (62.4%) of the respondents preferred sitting on a chair to breastfeed. The main reasons for positions adopted during breastfeeding were for comfort of mother/baby (60.8%) and convenience (29.5%). A majority (80.4%) of the respondents mostly practiced cross-cradle breastfeeding position while 13.3% practiced football hold. The rates of breast-to-baby and baby-to-breast latch-on practices were 18.0% and 41.3% respectively. A majority (75.7%) of the respondents reported that neck flexion, slight back flexion, arm support with pillow and foot rest is essential ergonomic posture while sitting to breastfeeding. Chi-square analyses of differences in breastfeeding knowledge, attitude and position practices are presented in Table 5. There was no significant association between breastfeeding posture practice and each of cumulative breastfeeding knowledge score levels (X^2^ = 0.044; p = 0.834) and attitude scores (X^2^ = 0.700; p = 0.403). However, there was a significant association between having previous training on breastfeeding and cumulative breastfeeding knowledge score level (X^2^ = 6.104; p = 0.013) while parity was significantly associated with breastfeeding posture practice (X^2^ = 10.022; p = 0.002).

**Table 4:** Breastfeeding postures, positioning, hold practice and latching techniques

**Variable**	**n**	**%**
Breastfeeding posture practice		
Advisable	273	71.3
Not advisable	110	28.7
Breastfeeding position practice		
Side-lying	47	12.3
Sitting on a mat	35	9.1
Sitting on the side of bed	62	16.2
Sitting on a chair	239	62.4
Reason for Breastfeeding position practice		
Comfort of the mother/baby	233	60.8
Convenience	113	29.5
Religion	8	2.1
No obvious reason	29	7.6
Breastfeeding hold practice		
Cradle hold	7	1.8
Football hold	51	13.3
Side-lying	17	4.5
Cross-cradle hold	308	80.4
Latch-on to breast practice		
Mothers who specified breast-to-baby	69	18.0
Mothers who specified baby-to- breast	158	41.3
Mothers who specified breast-to-baby and baby-to- breast	113	29.5
Mothers who gave no specific answer	43	11.2
Essential ergonomic posture while sitting to breastfeeding		
Slight neck flexion	33	8.6
Slight back upper back flexion	11	2.9
Foot rest	26	6.8
Arm support (pillow)	23	6.0
All of the above	290	75.7

**Table 5:** Chi-square analyses of differences in breastfeeding knowledge, attitude and position practices (N = 383)

	**Knowledge of breastfeeding**			
	**Good n (%)**	**Poor n (%)**	**X**^**2**^	**p-value**
Parity				
Primiparous	98(74.2)	34(25.8)	0.864	0.353
Multiparous	175(69.7)	76(30.3)		
Previous training on breastfeeding				
Yes	195(71.4)	71(28.6)	6.104	0.013
No	71(64.5)	46(35.5)		
	**Attitude towards breastfeeding**			
	**Good**	**Poor**	**X**^**2**^	**p-value**
Parity				
Primiparous	71(53.8)	61(46.2)	0.088	0.766
Multiparous	139(55.4)	112(44.6)		
Previous training on breastfeeding				
Yes	144(54.1)	122(45.9)	0.003	0.958
No	63(53.8)	54(46.2)		
	**Breastfeeding posture practice**			
	**Advisable**	**Not advisable**	**X**^**2**^	**p-value**
Parity				
Primiparous	111(84.1)	21(15.9)	10.022	0.002
Multiparous	236(94.0)	15(6.0)		
Previous training on breastfeeding				
Yes	251(94.4)	15(5.6)	1.818	0.178
No	106(90.6)	11(9.4)		
Knowledge of breastfeeding				
Good	254(71.2)	103(28.8)	0.044	0.834
Poor	19(73.1)	7(26.9)		
Attitude towards breastfeeding				
Positive	195(94.2)	12(5.8)	0.700	0.403
Negative	162(92.1)	14(7.9)		

## Discussion

This study assessed breastfeeding knowledge, attitude and practices of postures, positioning, hold techniques and latch-on among Nigerian mothers. From the result, there was a high awareness of EBF and full breastfeeding among the mothers. This finding is consistent with previous reports from Nigeria that showed high rates of awareness of EBF among working class mothers and women who have no paid occupation outside the home and instead focus their lives on motherhood [23,30,31]. The high level of awareness about EBF and full breastfeeding in this study may be attributed to the baby-friendly initiative programme being practiced at the centres where the study was conducted. From this study, information regarding breastfeeding was gotten mostly from hospitals. In line with this result, several studies have proved that baby-friendly hospitals are important channels for dissemination of knowledge about breastfeeding [27,32,33]. According to Bartington et al. [34] mothers who delivered in baby friendly maternity hospital units are more likely to initiate breastfeeding than those who did not deliver in baby friendly hospitals.

This study’s findings on the knowledge of the mothers regarding breastfeeding revealed that majority of the women in this study had good knowledge about breastfeeding. This finding confirms previous results that Nigerian mothers have good knowledge of EBF [24-26,35]. In consonance with literature on knowledge about breastfeeding, most of the mothers in this study agrees that breastfeeding promotes mother-baby bonding [36,37], frequent breastfeeding in the early period can help reduce jaundice [38,39], and that growth pattern of breastfed infants differ from those of formula fed [40-42]. The mothers in this study consider three month maternity leave duration as insufficient to encourage EBF and advocated for policies on longer leave for new mothers and availability of designated child care centers at workplaces to facilitate successful breastfeeding. A previous study reported dissatisfaction of mothers with the length of maternity leave and lack of right environment for breastfeeding at workplaces among Jordanian women [27]. Ong et al. [43] reported that inadequate facility for breastfeeding at workplace significantly reduces the duration of breastfeeding among working mothers. Similarly, extensions of maternity leave up to the first six month of child’s age to achieve optimal level of EBF practices was advocated in another study among Ethiopian women [44]. In order to promote optimal duration for EBF, the WHO advocated for minimum enabling conditions such as paid maternity leave, part-time work arrangements, facilities for expressing and storing breast milk and breastfeeding breaks for women in paid employment [45].

From this study, more than fifty percent of the women had positive attitude towards breastfeeding. Studies have found a direct correlation between positive attitude to breastfeeding and optimal EBF practice [46-48]. Positive parental attitudes towards infant feeding are reported to be an important component in child nutritional health [49]. In evaluating attitudes towards breastfeeding, perception of breastfeeding as easier than feeding infant formula and the roles of community and health workers in encouraging breastfeeding over feeding infant formula were the most rated influencing factors in this study. Other studies have implicated several factors for the different attitudes put forward by mothers towards breastfeeding. These include partner or friend/family support [48], fear of inadequate milk supply [25], ambivalent link of breastfeeding with sagging breast [50,51] and socio-cultural and psychological factors [52].

Compared with empirical findings, there are several anecdotal submissions and expert opinions on breastfeeding techniques which are often unreferenced or published as non-refereed materials. However, some previous studies have described breastfeeding techniques and habits; and optimal positions to stimulate breastfeeding and minimize fatigue [53-56]. To our knowledge, this study provided the first empirical analysis of techniques of breastfeeding posture, positioning of mother and baby, hold techniques and latch-on to breast or attachment practices among nursing mothers in SSA. From this study, most of the mothers were adjudged to be practicing advisable breastfeeding postures based on the pictorial self-report assessment of breastfeeding practice. However, controversies exist about the ideal sitting position, as there have been different recommendations over the years as regards the best sitting posture. More so, there is lack of consensus on the best breastfeeding postures. This present study chooses to designate conformity with recommendations on good sitting in literature as “advisable breastfeeding posture”. Considering there is no empirical evidence on the correct or standard breastfeeding posture in literature. However, Claus et al. [57] submitted that the best sitting posture should involve a minimal amount of stress and strain and should be conducive to maximize efficiency in the use of the body part. Therefore, this study conceived breastfeeding posture as the position assumed by the mothers for the purpose of breastfeeding. Nonetheless, sitting postures that deviate from standard recommendations for the workplace was deemed inappropriate for breastfeeding. Mbada et al. [58] submitted that inappropriate postures for prolonged time, as it is the case in breastfeeding, could lead to end range loading of periarticular structures and result in mechanical deformation of normal soft tissues. However, breastfeeding posture practice was not influenced by mothers’ knowledge and attitude regarding breastfeeding.

Unfortunately, there is an apparent dearth of empirical studies to compare this finding on posture practice in breastfeeding. A significant limitation of this study’s finding on posture practice in breastfeeding is the heterogeneity of the sample. Expected postures would be quite different if babies were currently 6 months old or currently 18 months old, as a result, the age of the child and the experience of the mother become important determinants.

Sitting on a chair to breastfeed was the most preferred breastfeeding position by mothers in this study and it was reportedly due to comfort of mother/baby and convenience. Sitting on the bedside to breastfeed was the second most utilized breastfeeding position while breastfeeding in side-lying was the least utilized position. Sitting upright in a breast-to-baby latch-on and holding baby in a cradle, cross-cradle or football position is considered as the mainstream and traditional approach in breastfeeding [59,60]. Some breastfeeding experts may traditionally have insisted on upright sitting postures because of etiquette, leaning back perhaps being associated with slouching or an unkempt appearance [59] and also that sitting upright for breastfeeding allows the women’s breasts to ‘point downwards and outwards’ allowing for attachment of baby to the breast [61]. However, sitting upright is believed to be fraught with potential for muscular fatigue and the most difficult body posture to sustain [62]. Furthermore, upright sitting breastfeeding position with no support for the back and may overstress the musculature of the trunk muscles [58]. Inadvertently, mothers are often advised to initiate breastfeeding in side-lying especially after caesarean section [59]. Additionally, breastfeeding in side-lying is thought to be comfortable and relaxing for the mother and child. It is adduced that the infrequent use of side-lying position by mothers in this study may be associated with certain believes that the position is problematic to latch the baby on to the breast [61,63], erroneous fears of infection from breast milk dripping to the baby’s ears and possible risk of smothering and suffocation of baby when sleeping especially by obese mothers or those with large breasts.

Cross-cradle hold was the most reported baby hold techniques practiced by the mothers in this study. This finding is consistent with several non-refereed expert opinions. This breastfeeding hold-position is believed to allow for more control of the infant’s head with just one arm while the mother put the breast in the mouth of the infant [64,65]. Football hold was also reported in this study as the second most practice hold technique among the mothers. This position is similar to tucking the baby under the arm like a football with the baby’s feet extending across the body. The baby’s head and shoulders are supported with the hand on the same side of the feeding breast [65]. It is important to note that breastfeeding hold is a dynamic practice, as mothers would constantly experiment with various positions and holds for their comfort and that of the child. However, this study sought mothers’ assessment of their most usual breastfeeding hold practices. Although, mixed and variable breastfeeding position practices are common among mothers.

Baby-to-breast latch-on or attachment was the most common practice among the mothers. The latch-on position of baby on the breast has been reported to be a very important process in breastfeeding and in prevention of nipple soreness [66]. Teicher [67] submitted that the ability of the baby to actually obtain milk at breast is linked to baby-led latching; maternal positioning of herself and her baby for comfort. Baby-led latching which involves bringing the baby’s body to the breast, adjusting mother or baby’s position, and other techniques are important in resolving latch problems [67]. Therefore, mothers understanding of how to facilitate adequate contact between the baby’s mouth and the mother’s breast and how to recognize poor positioning are important areas of research [68]. Furthermore, the result of this study showed that majority of the mothers demonstrated a level of knowledge of breastfeeding ergonomics which include the use of arm support and foot rest and the posturing of the neck and back. Fitting the act of breastfeeding to the body through the use of supports and good postural alignment of the spine is ergonomic. These can help prevent breastfeeding related musculoskeletal disorders resulting from the effect of poor postures and positioning on joints, ligaments, and muscles. However, the framing of questions on ergonomics in this study did not allow the opportunity to choose more than one option. Future studies could explore a multiple choice or open ended assessment of ergonomic practices of breastfeeding mothers. Many mothers may have thought that footstools and pillows are advisable, but the busy and mobile lives of many mothers may have limited the practice.

## Conclusion

Nigerian mothers demonstrated good knowledge and positive attitude towards breastfeeding. Breastfeeding was mostly believed to promote mother-baby bonding. Increasing length of time for maternity leave and providing designated areas at work places is believed to facilitate breastfeeding. Most of the mothers practiced advisable breastfeeding postures, preferred sitting on a chair to breastfeed and utilized cross-cradle hold and baby-to-breast latch-on.

## Competing interests

The authors declare that they have no competing interests.

## Authors’ contributions

CEM conceived the idea for this study, participated in the design of methodology, data collection and analysis, interpretation of data and prepared the final manuscript for publication. AEO, JOF, FAF, AOO, TOA, AAO and OAA participated in the design of the study’s methodology, interpretation of data and drafted the manuscript. FCO-A participated in the design of the study’s methodology and data acquisition and drafted the manuscript. All authors read and approved the final manuscript.
